# Effects of quercetin on spatial memory, hippocampal antioxidant defense and BDNF concentration in a rat model of Parkinson’s disease: An electrophysiological study

**DOI:** 10.22038/AJP.2021.18526

**Published:** 2021

**Authors:** Mehrdad Naghizadeh, Mohammad Ali Mirshekar, Farzaneh Montazerifar, Saeideh Saadat, Ali Shamsi Koushki, Saber Jafari Maskouni, Maryam Afsharfar, Saiedeh Arabmoazzen

**Affiliations:** 1 *Student Research Committee, Zahedan University of Medical Sciences, Zahedan, Iran*; 2 *Department of Food Sciences and Nutrition, School of Medicine, Zahedan University of Medical Sciences, Zahedan, Iran*; 3 *Clinical Immunology Research Center, Zahedan University of Medical Sciences, Zahedan, Iran*; 4 *Department of Physiology, School of Medicine, Zahedan University of Medical Sciences, Zahedan, Iran*; 5 *Deputy of Research and Technology, * *Zahedan University of Medical Sciences, Zahedan, Iran *

**Keywords:** Quercetin, Parkinson’s disease, Spatial memory, Oxidative stress

## Abstract

**Objective:**

Quercetin is one of the most popular flavonoid with protective effects against neural damages in Parkinson's disease (PD). We assessed the effect of quercetin administration on memory and motor function, hippocampal ‎ oxidative stress and brain-derived neurotrophic factor (BDNF) level in a 6-OHDA-induced Parkinson's rat model.

**Material and Methods:**

The animals were divided into the following five groups (n=8): control, sham-surgery (sham), lesion (PD), and lesion animals treated with quercetin at doses of 10 (Q10) and 25 (Q25) mg/kg. For induction of a model of PD, 6-OHDA was injected into the striatum of rats. The effects of quercetin were investigated on spatial memory, hippocampal BDNF and malondialdehyde (MDA) levels, and total antioxidant capacity (TAC). Spatial memory was assessed by Morris water maze test, and the neuronal firing frequency in hippocampal dentate gyrus (HDG) was evaluated by single-unit recordings.

**Results:**

Mean path length and latency time, rotational behavior and hippocampal MDA concentration were significantly increased, while time spent in the goal quadrant, swimming speed, spike rate, and hippocampal levels of TAC and BDNF were significantly decreased in the PD group compared to the sham group (p<0.01 to p<0.001). Quercetin treatment significantly enhanced time spent in goal quadrant (p<0.05), swimming speed (p<0.001) and spike rate (p<0.01), improved hippocampal TAC (p<0.05 to p<0.001) and BDNF (p<0.01 to p<0.001) level, and decreased mean path length (p<0.001), latency time (p<0.05 to p<0.001), rotational behavior and hippocampal MDA concentration (p<0.05).

**Conclusion:**

The cognitive-enhancing effect of quercetin might be due to its antioxidant effects in the hippocampus.

## Introduction

Parkinson’s disease (PD) as one of the most common progressive neurodegenerative diseases, is associated with inadequate dopamine concentration in the corpus striatum and it causes a regular dysfunction of the basal nuclei in midbrain (Shim et al., 2009 ▶). Disorders of the basal ganglia such as PD are commonly thought of as motor disorders primarily; however, the cognitive symptoms of these diseases such as executive dysfunction, and learning, memory and attention deficits are prominent and often more disabling than the hallmark motor symptoms. Cognitive symptoms are associated with dysfunction in cholinergic circuits, in addition to the abnormalities in the prefrontal dopaminergic system (Narayanan et al., 2013 ▶). To date, the precise mechanism responsible for pathogenesis of PD is not well understood. However, inflammation, apoptosis, and oxidative stress are critical factors associated with the death of dopaminergic neurons (Hu et al., 2010 ▶).

Oxidative stress plays a key role in the degeneration of dopaminergic neurons in PD. Disruptions in the physiologic maintenance of the redox potential in neurons interfere with several biological processes, ultimately leading to cell death (Guo et al., 2018 ▶). Studies have suggested the potential involvement of the sources and mechanisms such as dopamine metabolism, mitochondrial dysfunction, neuroinflammation, iron, calcium, and aging in overwhelming oxidative stress and neurodegeneration that occur in PD (Chang and Chen, 2020 ▶).

Brain-derived neurotrophic factor (BDNF), as a neurotrophic factor, is a key molecule involved in plastic changes related to learning and memory, and increases the tolerance of dopamine neurons against acidic environment (Wang et al., 2019 ▶; Rahmani et al., 2019 ▶). Signaling pathway of BDNF/TrKB plays a major role in memory processes. Changing in the expression of BDNF in specific groups of neurons can result in depression, Alzheimer’s disease, epilepsy, Huntington and PD (Cattaneo et al., 2005 ▶; Russo-Neustadt and Chen, 2005 ▶).

It was found that injection of 6-hydroxydopamine (6-OHDA) into the striatum of rats creates a model of PD (Lev et al., 2013 ▶). It has been reported that 6-OHDA causes some of the pathological, biochemical and behavioral changes into the striatum (Schober, 2004 ▶). The most important defects can be disorders in spatial memory, working memory, sustained attention and cognitive flexibility in experimental models of Parkinson (De Leonibus et al., 2007 ▶; Arbabi et al., 2016 ▶; O’Neill et al., 2007 ▶).

Diet and supplement therapy have shown usefulness in improving symptoms of PD (Barichella et al., 2009 ▶). Applications of antioxidants to modulate oxidative stress could be a strategy in treating PD. Quercetin is a potent antioxidant flavonoid found in common vegetables and fruits. Previous studies have demonstrated that quercetin has many good biological properties for human health including antioxidant (Sriraksa et al., 2012 ▶) and anti-inflammatory activities (Amália et al., 2007 ▶; Huang et al., 2012 ▶). It is suggested that cognitive-enhancing effect of quercetin occurs partly because of decreased oxidative damage resulting in increased neuron density (Sriraksa et al., 2012 ▶). Quercetin exhibited neuroprotective effect againsts oxidative damage induced by 6-OHDA (Haleagrahara et al., 2011 ▶). Quercetin also up-regulated mitochondrial complex-I activity to protect against programmed cell death in a rotenone model of PD in rats (Karuppagounder et al., 2013 ▶). Moreover, it was found that quercetin attenuated neuronal death in the hippocampus resulting in improved learning and memory in arm maze test (Pu et al., 2007 ▶).

Based on the antioxidant and anti-inflammatory activities of quercetin, it seems that it may improve the oxidative balance and spatial memory in PD. To clarify this issue, the present study aimed to determine the effect of quercetin on spatial memory, hippocampal total antioxidant capacity (TAC), and levels of malondialdehyde (MDA) and BDNF in a 6-OHDA-induced Parkinson's rat model. Moreover, the neuronal firing frequency in the hippocampal dentate gyrus (HDG) was evaluated by single-unit recordings.

## Materials and Methods

**Animals**

The current study was conducted on 40 adults male Wistar rats (body weight 200-230 g). All of the rats were housed in standard cage (2 rats per cage), under constant condition of temperature (20±2°C), humidity (15-20%) and lighting (12 hr light: 12 hr dark cycle) with free access to food and water *ad libitum*. The experimental protocols were approved by the ethical committee on animal experiments of the Research Council of Zahedan University of Medical Sciences, Zahedan, Iran, in agreement with National Institute of Health Guide for care and use of laboratory animals (ethical number: IR.ZAUMS.REC.1398.389).

**Experimental design**

The animals were randomly divided into five groups (8 rats in each group) as follows: 1. Control group without surgery. 2. Sham-surgery group (sham) which received 0.2% ascorbate-saline solution via an intra- striatal injection. 3. Lesion group (PD) which received 8 μg of 6-OHDA dissolved in 3 μL normal saline (NS) with 0.2% ascorbic acid via an intra- striatal injection. 4 & 5. Lesion animals which received 10 (Q10) and 25 (Q25) mg/kg of quercetin dissolved in 1 ml of NS by gavage, once daily for a period of one month after the unilateral lesion of right striatum was induced by 6-OHDA (Rahmani et al.). All chemical substances were purchased from Sigma Chemical Co (St. Louis, MO).

**Stereotaxic surgery**

The rats were anaesthetized by intraperitoneal (i.p.) administration of ketamine hydrochloride/xylazine (90/10 mg/kg), and each rat was placed in a stereotaxic stand. To model PD, 6-OHDA was injected slowly using Hamilton syringe into the right striatum (anteroposterior −4.4 mm from bregma, mediolateral 1.3 mm from midline, and dorsoventral 8.4 mm from the skull, according to the atlas of Paxinos and Watson). 

**Apomorphine rotation test**

The rats were tested for rotational behavior by apomorphine hydrochloride (Sigma Chemical Co), 3 weeks after the surgery. First, each rat was placed in a cylindrical container (diameter 33 cm and height 35 cm) to adapt to the test environment for 10 min. One minute after injection of apomorphine (0.5 mg/kg, i.p), the rats were placed in a cylindrical container and full contralateral rotations were counted. The number of contralateral rotations (opposite to the lesion) was monitored for 30 min. The number of pure turns was calculated by subtraction the rotations in the opposite direction from the rotations in the direction of the damage. This test was performed twice and rotations were recorded by video camera.

**Morris water maze (MVM) test**

The MWM task consisted of a black metal pool (180 cm in diameter × 60 cm tall) filled with water (26±1°C). The pool was divided into 4 quadrants (NE, NW, SE, and SW). A removable platform (12 cm in diameter and 38 cm in height) was placed 2 cm below the water surface. The platform was located in the target quadrant. To identify spatial orientation and learning, signs were mounted in four directions on the walls of the pool. Each animal was randomly placed in the water along the maze wall in one of the 4 starting points (NE, NW, SE and SW) and it was allowed to swim freely until it found and climbed onto the platform. If the animal did not find the platform within 90 sec, manual guidance was provided to locate the platform and then, the animal rested on the platform for 30 sec. For spatial learning, the animals were trained in the water maze for 4 days and 4 training sessions per day, and the animal's performance in the maze was transmitted to a computer via an infrared camera. The performance of memory was evaluated 24 hr after the training process by performing a 60-second recall test. At this stage, after removing the platform, the animal was released from the opposite side of the platform into the water to find the platform. Time elapsed and distance traveled to find the platform on training days as well as time to reach the platform, mileage, and percentage of time spent in the target area, were evaluated in the recall test.

**Electrical recording from HDG**

Single-unit recordings provide a method of measuring the electro-physiological responses of a single neuron using a microelectrode system. To evaluate neuronal firing in the HDG area, a single unit recording method was used. Briefly, an extracellular recording of a single neuron was taken by the Tungsten Microelectrode (WPI; with an extra-fine tip; 1MΩ impedance tip) in urethane (1.5 g/kg; i.p.)-anesthetized rats. The microelectrode was implanted using a manual microdrive into the dentate gyrus (DG) cells according to the Paxinos stereotactic coordinates until the maximum spike activity of neurons with a signal/noise ratio of more than 2 isolated from the contextual noise was achieved (Paxinos and Watson, 2006 ▶). Band pass was filtered at 0.3–3 kHz and digitized at 50 kHz sampling rate and 12-bit voltage resolution via a data acquisition system. The action potentials were evaluated by window discriminator software based on the spike amplitude. The numbers of spikes/bin were calculated over 20 min in all experimental groups.

**Biochemical analysis**

Animals were sacrificed, and hippocampal tissues were isolated and homogenized in cold phosphate-buffered saline (PBS). The homogenates were centrifuged at 1000 g at 4°C for 10 min. The supernatants were stored at −70°C for measurement of MDA, TAC and BDNF levels.

**Determination of MDA, TAC and BDNF levels**

MDA level in the supernatant was measured as described before (Mirshekar et al., 2018 ▶). Briefly, trichloroacetic acid and TBARS (thiobarbituric acid reactive substances) reagent were added to supernatant, then mixed and incubated in boiling water for 90 min. After cooling, samples were centrifuged at 1000 g for 10 min. Light absorbance of samples was read at 534 nm wave-length by a spectrophotometer (Biowave II, UK), and MDA concentration is reported as nM/mg protein.

Hippocampal TAC was measured with the use of a commercial kit (ZellBio GmbH, Germany) following the manufacturer’s instructions on the basis of the oxidation reduction colorimetric assay at a wavelength of 490 nm. TAC level was considered the amount of antioxidant in the sample that was comparable to ascorbic acid action as a gold standard. This method can determine TAC with 0.1 mM sensitivity.

The concentration of BDNF in the hippocampal tissues was determined by ELISA methods and using commercial kits (ZellBio, GmbH, ‎Germany), using the instructions provided by the kits. The absorbance of the samples and standards were read using a microplate reader.

**Statistical analysis**

Data were analyzed using Graph Pad Prism 7.0 (Graph Pad Software, San Diego, CA) and are expressed as the mean±SEM in all experiments. one-way ANOVA was used for analyze MDA, TAC, BDNF concentration, apomorphine-induced rotation and average of spike /Time but MWM data were analyzed by Two-way ANOVA repeated measurements, followed by Tukey multiple comparison test for statistical comparisons. In this study, statistical significance was set at p≤0.05.

## Results

**Effect of quercetin on apomorphine-induced rotation in the 6-OHDA-induced Parkinson's rat model**

**Figure 1:**
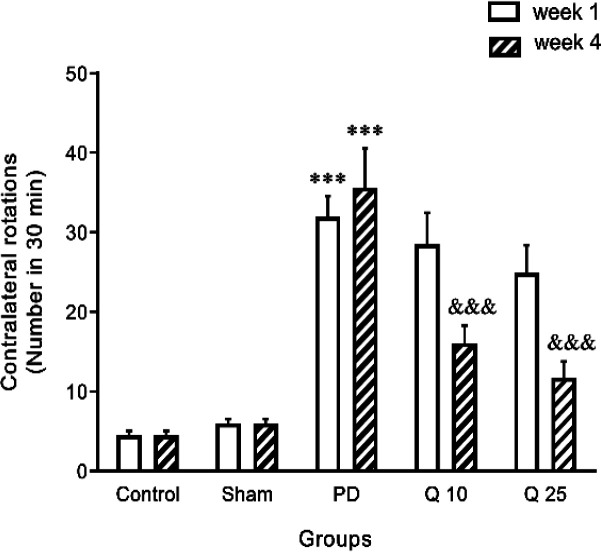
The effect of quercetin on apomorphine-induced rotation in the 6-OHDA-induced Parkinson's rat model in the control, sham, Parkinson's disease (PD) and quercetin 10 (Q10) and 25 mg/kg (Q25)-treated groups (***p<0.001, PD vs. sham and control groups, and &&& p<0.001, Q10 and Q25 vs. PD group). Two-way ANOVA repeated measurements, followed by Tukey’s *post hoc* tests

Animals showed contralateral rotational behavior following apomorphine injection 1 and 4 weeks after unilateral administration of 6-OHDA. Apomorphine caused a significant increase in the contralateral rotating in the 6-OHDA-treated groups compared with the control and sham groups (F (1, 7) = 78.67; p<0.001 in the 1st and 4th week). Quercetin administration (10 and 25 mg/kg) significantly decreased the number of rotations (F (4, 28) = 59.87; p<0.001) in the fourth week (Figure 1).

**Effect of quercetin on performances of the rats in MWM**

**Figure 2:**
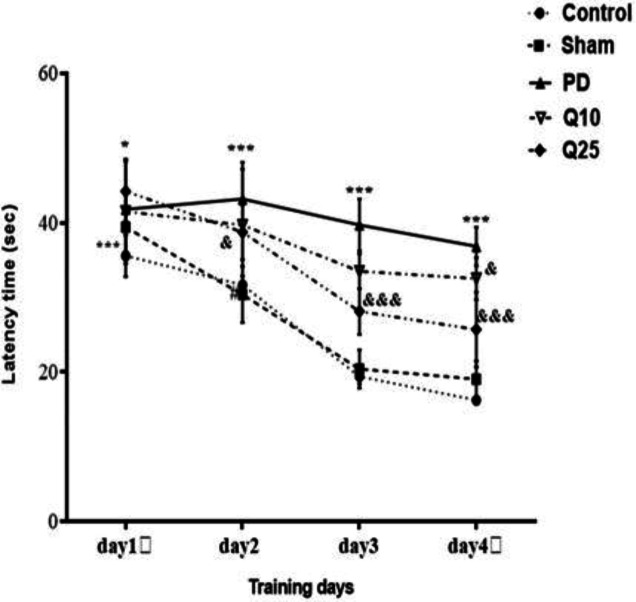
The effect of quercetin on escape latency in the 6-OHDA-injected memory impairments in the control, sham, Parkinson's disease (PD) and quercetin 10 (Q10) and 25 mg/kg (Q25)-treated groups (*p<0.05, ***p<0.001, PD vs. sham and control groups, and & p<0.05, &&& p<0.001, Q10 and Q25 vs. PD group). Two-way ANOVA repeated measurements, followed by Tukey’s *post hoc* tests

**Figure 3:**
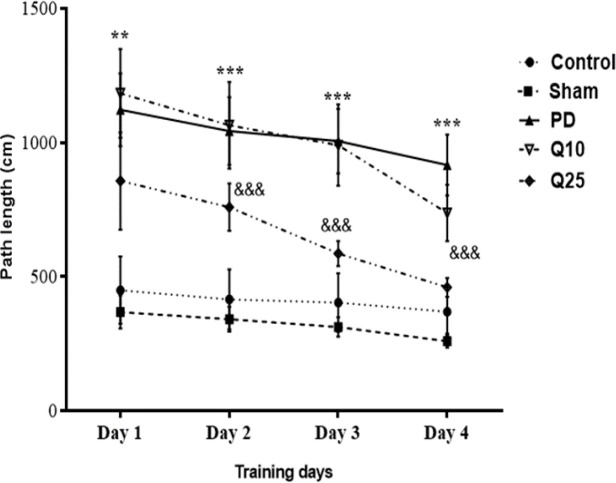
The effect of quercetin on path length on 6-OHDA-induced memory impairment in the control, sham, Parkinson's disease (PD) and quercetin 10 (Q10) and 25 mg/kg (Q25)- treated groups (**P<0.01, ***p<0.001, PD vs. sham and control groups, and &&& p<0.001, Q10 and Q25 vs. PD group). Two-way ANOVA repeated measurements, followed by Tukey’s *post hoc* tests

The mean escape latencies of PD group significantly increased compared to the sham group [F (12, 84) = 1.285; p<0.001]. After quercetin administration, a significant decrease was shown in the mean escape latencies of quercetin-treated groups (10 and 25 mg/kg**) **compared to the PD group [F (12, 84) = 1.285; p<0.05 and p<0.001] (Figure 2). The mean path length on days 1 [F (12, 84) = 2.365; p<0.01], 2, 3 and 4 [F (12, 84) = 2.365; p<0.001] significantly increased in the PD group compared to the sham group. Interestingly, a significant decrease was observed in the mean path length in the quercetin-treated groups (especially 25 mg/kg) compared to the PD group [F (12, 84) = 2.365; p<0.001] (Figure 3). 

**Figure 4:**
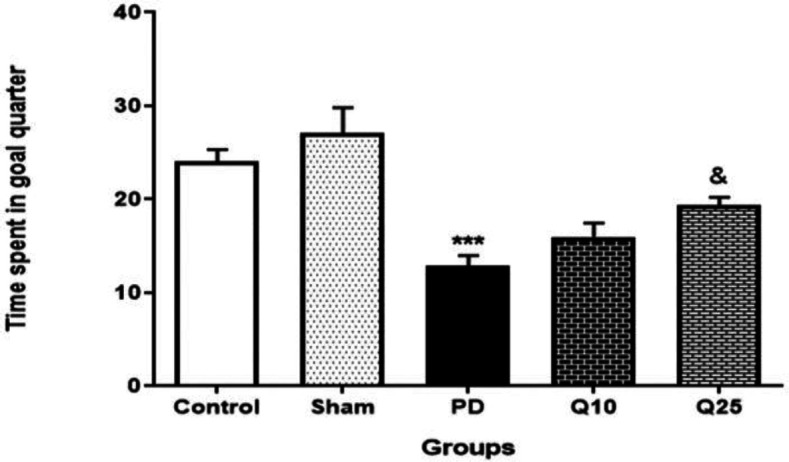
The effect of quercetin on the percent of total time spent by each rat in the goal quarter in 6-OHDA-injected memory impaired animals in the control, sham, Parkinson's disease (PD) and quercetin 10 (Q10) and 25 mg/kg (Q25)- treated groups (***p<0.001, PD vs. sham and control groups, and & p<0.05, Q10 and Q25 vs. PD group). Two-way ANOVA repeated measurements, followed by Tukey’s *post hoc* tests

The time spent in the goal quadrant (TSGQ) in the PD group was significantly lower than the sham group [F (4, 35) = 12.64; p<0.001]. Quercetin administration (25 mg/kg) significantly increased the time spent in the goal quadrant [F (4, 35) = 12.64; p<0.05] (Figure 4).

**Figure 5:**
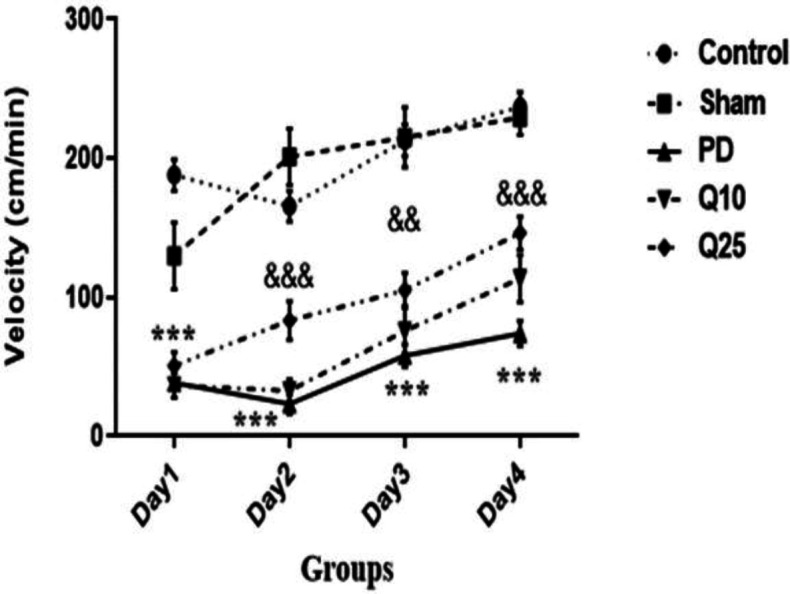
The effect of quercetin on speed on 6-OHDA-induced memory impairment in the control, sham, Parkinson's disease (PD) and quercetin 10 (Q10) and 25 mg/kg (Q25)-treated groups, (***p<0.001, PD vs. sham and control groups, and && p<0.01, &&& p<0.001, Q10 and Q25 vs. PD group). Two-way ANOVA repeated measurements, followed by Tukey’s *post hoc* tests

The swimming speeds in the PD group were significantly decreased compared to the control and sham groups [F (12, 105) = 2.7; p<0.001]. Swimming speed was significantly increased in quercetin-treated groups compared to the PD group [F (12, 105) = 2.7; p<0.001] (Figure 5).

**Effect of quercetin on the HDG neuronal firing rate**

**Figure 6:**
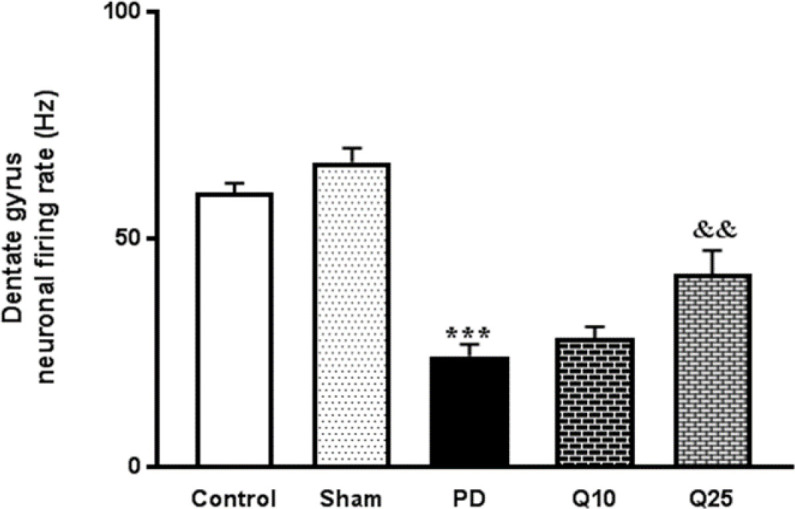
The effect of quercetin administration on the hippocampal dentate gyrus neuronal firing rate in the control, sham, Parkinson's disease (PD) and quercetin 10 (Q10) and 25 mg/kg (Q25)- treated groups (***p<0.001, PD vs. sham and control groups, and && p<0.01, Q10 and Q25 vs. PD group). one-way ANOVA repeated measurements, followed by Tukey’s *post hoc* tests

Representative tracings and magnification of a sample spike of the neuronal activity of granular cells in the DG from all groups are presented in Figure 6. The average number of spikes/bin was decreased in the PD group compared to the sham group (F (4, 35) = 32.8; p<0.001). Moreover, high-dose quercetin (25 mg/kg) increased the spike rate in comparison to the PD group (F; p<0.01).

**Effect of quercetin on MDA, TAC and BDNF levels in HDG tissue**

**Figure 7:**
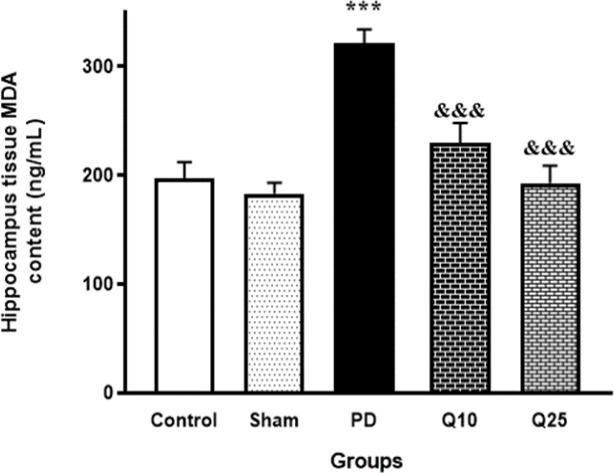
The effect of quercetin administration on hippocampal concentration of malondialdehyde (MDA) in the control, sham, Parkinson's disease (PD) and quercetin 10 (Q10) and 25 mg/kg (Q25)- treated groups (***p<0.001, PD vs. sham and control groups, and &&& p<0.001, Q10 and Q25 vs. PD group). one-way ANOVA repeated measurements, followed by Tukey’s *post hoc* tests

**Figure 8:**
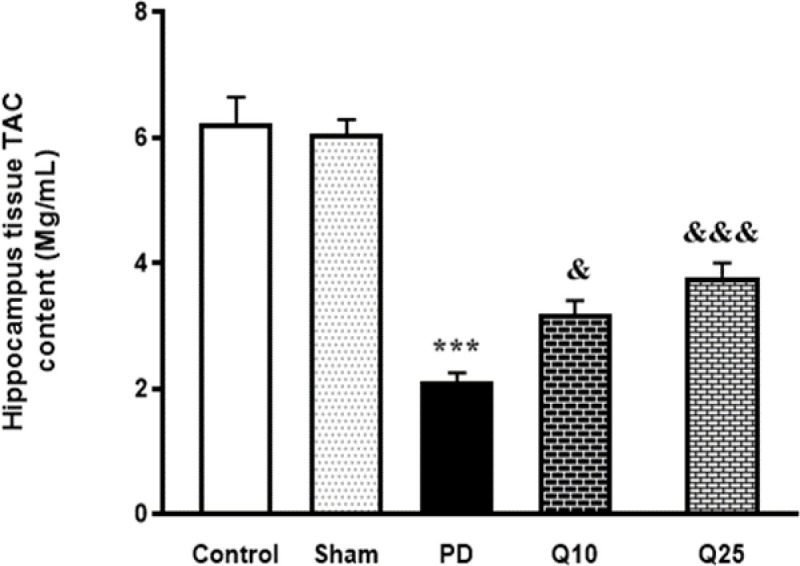
The effect of quercetin administration on total antioxidant capacity (TAC) content in the control, sham, Parkinson's disease (PD) and quercetin 10 (Q10) and 25 mg/kg (Q25)- treated groups (***p<0.001, PD vs. sham and control groups, and & p<0.05, &&& p<0.001, Q10 and Q25 vs. PD group). one-way ANOVA repeated measurements, followed by Tukey’s *post hoc* tests

Injection of 6-OHDA in the PD group, caused a significant increase in MDA concentration in HDG compared to the control and sham groups [F (4, 35) = 15.26; p<0.001]. In the groups treated with quercetin (10 and 25 mg/kg), significant reductions of increased MDA activity in HDG were found compared to the PD group [F (4, 35) = 15.26; p<0.05] (Figure 7). The TAC in the HDG tissue remarkably decreased in the PD group compared to the control and sham animals [F (4, 35) = 47.46; p<0.001]. Data showed that quercetin (10 and 25 mg/kg) significantly improved TAC in the HDG [F (4, 35) = 47.46; p<0.05 and p<0.001, respectively] (Figure 8).

**Figure 9:**
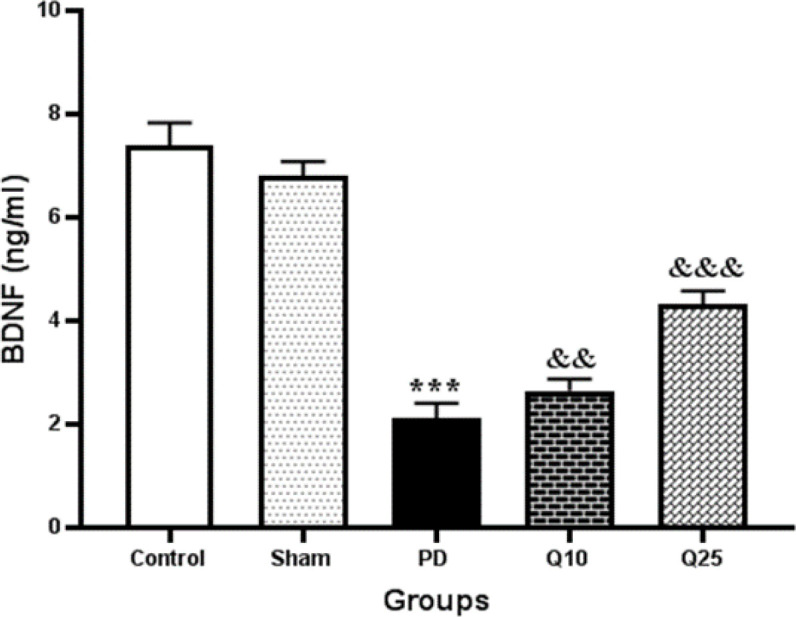
The effect of quercetin administration on hippocampal concentration of brain-derived neurotrophic factor (BDNF) in the control, sham, Parkinson's disease (PD) and quercetin 10 (Q10) and 25 mg/kg (Q25)- treated groups, (***p<0.001, PD vs. sham and control groups, and && p<0.01, &&& p<0.001, Q10 and Q25 vs. PD group). One-way ANOVA repeated measurements, followed by Tukey’s *post hoc* tests

The data showed that hippocampal BDNF concentration in the PD group was significantly lower than the control and sham groups (p<0.001). Quercetin administration (10 and 25 mg/kg) caused a significant increase in BDNF level compared to the PD group (p<0.01 and p<0.001, respectively) (Figure 9).

## Discussion

In this study, the protective effect of quercetin was investigated on memory and cognition, antioxidant defense‎, and hippocampal BDNF concentration in the 6-OHDA-induced Parkinson's rat model.

Rotation induced by apomorphine test was used as a standard method to monitor motor impairment and functional recovery in the 6-OHDA-induced hemi-PD model (Creese et al., 1977 ▶). In the present study, we used this test to confirm the induction of PD in rats. The data showed that 6-OHDA injection caused a significant increase in apomorphine-induced rotation. Previous studies demonstrated that intra-medial forebrain bundle (MFB) injection of 6-OHDA with reduced level of striatal dopamine, causes damage to the nigrostriatal dopaminergic system (Schwarting and Huston, 1996 ▶) and leads to cognitive deficits in animals (De Leonibus et al., 2007 ▶; Sriraksa et al., 2012 ▶).

Cognitive impairment is commonly observed in Parkinson patients. Accordingly, we investigated the effect of quercetin on spatial memory by using the Morris water maze test and recorded escape latency, path length, percentage of time spent and speed as the indices. Our results showed that mean swim path length and escape latency to find the hidden platform in the PD group significantly increased, whereas number of entries and time spent in the target quadrant decreased. These findings confirmed a memory disorder in PD rats.

It has been suggested that dentate gyrus spikes might be important for memory consolidation (Nokia et al., 2017 ▶). Disrupting DG spikes-related silencing of hippocampal CA1 pyramidal cell firing consistently after the training session impaired the learning of trace eyeblink conditioning, a Pavlovian conditioning task dependent on the hippocampus (Kim et al., 1995 ▶). Our study showed that induction of PD by 6-OHDA can decrease DG firing.

We found that quercetin significantly improved the 6-OHDA-induced cognitive impairment as indicated by the decrease of the mean escape latency and time needed for discovering the hidden platform area. These results are consistent with previous studies indicating that quercetin improves cognitive impairment (Prasad et al., 2013 ▶; Sriraksa et al., 2012 ▶). Compared to the doses of quercetin (100, 200, and 300 mg/kg) used in the previous study (Sriraksa et al., 2012 ▶), lower doses of quercetin improved spatial learning and memory function in the present study. The results of our study demonstrated that quercetin at a high dose significantly increased neuronal firing rate in the PD groups. It was observed that quercetin protects against progressive degeneration of nigrostriatal, behavioral deficits and strata dopamine depletion (Ay et al., 2017 ▶). As a result, quercetin can effectively activate protein kinase D1 pro-survival signaling in dopaminergic cells. Moreover, quercetin treatment increases mitochondrial biogenesis in dopaminergic neurons. Quercetin protects against dopaminergic neurodegeneration in the MitoPark transgenic mouse model of PD (Ay et al., 2017 ▶). 

Our results indicated that 6-OHDA-induced PD reduces BDNF level in the hippocampus. The changes in BDNF level were shown to directly affect memory performance in animal models of neurodegenerative/neuropsychiatric diseases and in normal conditions (Miranda et al., 2019 ▶). In the present study, treatment with quercetin increased hippocampal BDNF level in the PD groups. The results of Rahvar et al. showed that quercetin (20 and 50 mg/kg) can increase the expression of BDNF and its mRMA in the hippocampus (Rahvar et al., 2018 ▶). Previous studies demonstrated that treatment with quercetin increases BDNF level and prevents rise of caspase-3 activity and cytochrome C level (Ola et al., 2017 ▶). Quercetin augments cAMP response element-binding protein (CREB) signaling and BDNF levels. Therefore, it increases CREB phosphorylation and BDNF expression (Ay et al., 2017 ▶). Quercetin was shown to scavenge the polychlorinated biphenyl (PCBs)-induced reactive oxygen species (ROS), thereby preventing transmembrane tight functional proteins in the hippocampus and maintaining the level of steady-state, thus protecting the BDNF signaling molecules in hemostasis (Selvakumar et al., 2018 ▶).

Oxidative stress has been reported to induce cognitive impairment in Parkinson's disease. Reports have showed the possible causal mechanism of neurotoxicity induced by 6-OHDA being related to oxidative stress. 6-hydroxydopamine can produce intracellular H_2_O_2_. This leads to the production of reactive hydroxyl radicals, increment of MDA level, and reduction of antioxidant enzymes activities (Kumar et al., 1995 ▶) as observed in the current study. Additionally, in this study, quercetin treatment increased TAC, and decreased MDA level in the hippocampal area. Our results suggest that the cognitive-enhancing effect of quercetin might be due to its antioxidant effect in the hippocampus. In this study, better results were observed in the high-dose-treated group because the active metabolites of quercetin might be able to reach to therapeutic level.

The results indicated that quercetin has an improving effect on 6-OHDA-induced disorder, and a protective action against neurotoxin-induced damage and behavioral deficits in rats. Quercetin probably restores neurological behaviors and improves spatial memory in the PD rat model by increasing the neuronal firing rate, modulating the neuronal oxidative stress, and maintaining the antioxidant status. Accordingly, it can be concluded that quercetin may be a potential flavonoid against the development of PD. However, further investigation is needed to clarify the mechanism of quercetin neuroprotective effects.
